# Effects of some flavonoids on the mycotoxin citrinin reduction by *Monascus aurantiacus* Li AS3.4384 during liquid-state fermentation

**DOI:** 10.1186/s13568-020-0962-7

**Published:** 2020-02-03

**Authors:** Yanling Wang, Heng Gao, Jianhua Xie, Xiujiang Li, Zhibing Huang

**Affiliations:** 1 State Key Laboratory of Food Science and Technology, Nanchang University, No. 235 Nanjing East Road, Nanchang, 330047 China; 2 Sino–German Joint Research Institute, Nanchang University, No. 235 Nanjing East Road, Nanchang, 330047 China; 3 The First Affiliated Hospital of Nanchang University, Nanchang University, No. 17 Yongwai Main Street, Nanjing West Road, Nanchang, 330006 China

**Keywords:** *Monascus*, Citrinin, Pigment, Genistein, Flavonoids

## Abstract

*Monascus* can produce many beneficial metabolites; however, it can simultaneously also produce citrinin, which seriously limits its application. Therefore, reducing the production of citrinin is of great interest. Herein, *Monascus aurantiacus* Li AS3.4384 (MAL) was used to optimize the liquid-state fermentation process and investigate the effects of genistein and other flavonoids on citrinin, pigments, and biomass of MAL. Results showed that citrinin decreased by 80%, pigments and biomass increased by approximately 20% in 12 days with addition of 20.0 g/L rice powder as a carbon source and 2.0 g/L genistein during shaking liquid-state fermentation. Further, genistein, daidzein, luteolin, apigenin, quercetin, baicalein, kaempferol myricetin, and genistin exerted different effects on citrinin production by MAL, with genistein causing the highest reduction in citrinin production during liquid-state fermentation, possibly due to the presence of C5-OH, C4′-OH, and C7-OH. Therefore, genistein can be added to the fermentation process of *Monascus* to reduce citrinin.

## Introduction

Since the era of Tang Dynasty (618–907 AD), *Monascus* has been used in China to produce sufu, wine, and colorants. Currently, *Monascus* is widely used in the fields of food, medicine and health care (Chen et al. 2017; Huang et al. 2019). *Monascus* can produce many useful secondary metabolites, such as *Monascus* pigments, Monacolin K, ergosterol, γ-aminobutyric acid, and unsaturated fatty acids (Akilandeswari and Pradeep 2016; Hilares et al. 2018; Li et al. 2019; Zhang et al. 2019; Gomes and Takahashi 2016). *Monascus* pigments have long been used as natural food additives, particularly in China, Japan, and other Southeast Asian countries (Dufosse 2018; Chen et al. 2015; Hajjaj et al. 1999). *Monascus* pigment is a mixture of polyketones belonging to the following three main pigments: red, orange, and yellow. Further, the six main pigment components include rubropunctamine and monascorubramine (red pigment), rubropunctatin and monascorubrin (orange pigment), and monascin and ankaflavin (yellow pigment) (Balakrishnan et al. 2013; Yongsmith et al. 2013; Lu et al. 2018; Schweiggert 2018; Lv et al. 2018). Studies have shown that some *Monascus* pigments also possess anti-inflammatory, anti-atherosclerosis, anti-cancer, anti-bacterial, and potential anti-obesity properties (Xu et al. 2018; Orak et al. 2018; Zheng et al. 2016).

*Monascus* secretes beneficial secondary metabolites while also producing a potentially toxic secondary metabolite, citrinin (Nigović et al. 2013). Citrinin is a mycotoxin of polyketides, and its main targeting organ is kidney. Reportedly, 50 μmol/L of citrinin can deregulate calcium homeostasis of PK15 cells, leading to cell death (Rumora et al. 2014). In the kidney and liver mitochondria, citrinin can inhibit activities of oxidoreductases and ATPases, interfere with the electron transport chain, reduce the efficiency of mitochondrial phosphorylation, and change the transmembrane potential, resulting in decreased intracellular respiratory efficiency. Protease activity is also blocked by citrinin. Thus, it seriously affects human health and hinders the development of *Monascus* products (Liao et al. 2014; Gayathri et al. 2015).

As a result, there is an urgent need to study methods to reduce citrinin production by *Monascus* spp. There are currently three approaches to investigate citrinin production. Firstly, a mutant strain can be generated to produce low-yield or non-producing citrinin-resistant strains (Dikshit and Tallapragada 2018). Jia et al.(2010) used traditional physical chemical mutagenesis and metabolic engineering methods to obtain a citrinin-free mutant of the gene *pksCT*, thereby obtaining a strain that does not produce citrinin. Secondly, medium optimization can reduce the yield of citrinin (Hu et al. 2016). Carels and Shepherd (1977) explored the effects of carbon source and nitrogen sources on citrinin and pigment. Wong et al. (1981) studied the effect of the carbon/nitrogen ratio on citrinin levels in the medium. Kang et al. (2014) proposed a method to reduce citrinin production and increase the yield of yellow pigment under low pH conditions. Tsukahara et al. (2009) have reported that increasing culture temperature can effectively increase the yield of pigment and reduce citrinin. Wan et al. (2017) proposed that citrinin production could be affected under a low frequency magnetic field. Thirdly, genetic engineering has been used recently to knock out key genes to reduce citrinin. Shimizu et al. (2007) significantly reduced citrinin production by knocking out the *ctnA* gene in the flanking region of the *pksCT* gene.

**Fig. 1:**
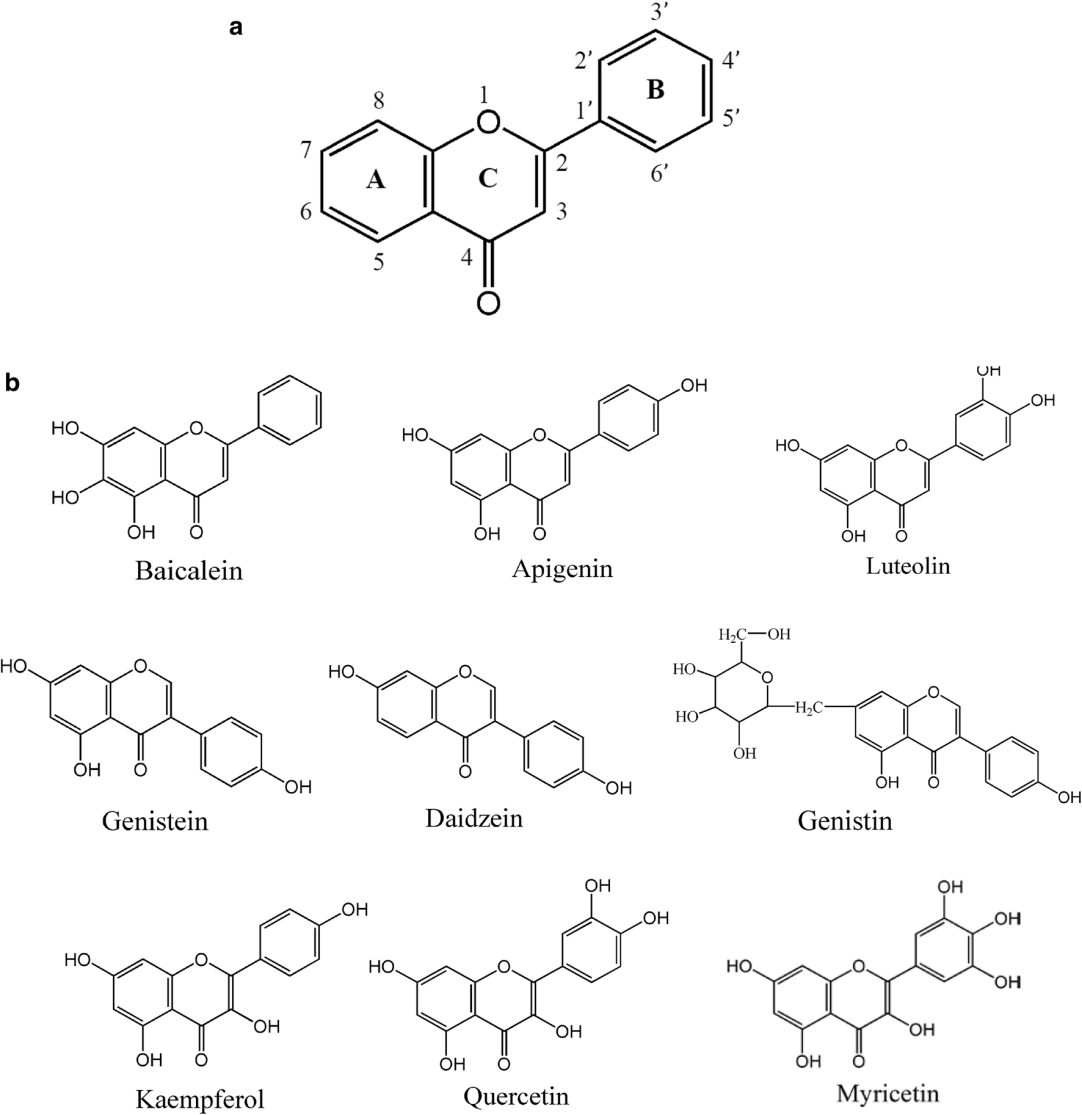
**a** Matrix structure of flavonoids, and **b** structural formulas of different flavonoids

Flavonoids are a class of compounds with a flavone structure. Flavonoids are mostly crystalline solids, with few as amorphous powders (Echeverry et al. 2010). They are widely distributed in the plant kingdom, and the fruits and vegetables that are consumed daily are rich in flavonoids. Although there are many kinds of flavonoids, their main structure is composed of two aromatic rings connected by an oxygen-containing heterocyclic ring (Fig. 1a). According to the difference of the substituents on the mother nucleus and the connection position of the B ring, the flavonoids can be divided into six categories: flavonols such as quercetin, myricetin, and kaempferol; flavonoids such as luteolin and baicalein; flavanols such as catechins; flavanones such as naringenin; anthocyanins, and isoflavones such as genistein (Wolfe and Liu 2008; Olagaray and Bradford 2019). Genistein is considered a preventive drug that can prevent various diseases, including cancer, postmenopausal bone loss, and cardiovascular disease. It is the most abundant isoflavone in soybean and has been reported to function as a protein tyrosine Kinase (PTK) inhibitor. It is also believed to becoming effective against antioxidants, to have anti-proliferative and anti-skin cancer functions, and to regulate estrogen function (Kundu et al. 2018; Wang et al. 2019; Fan et al. 2019). Flavonoids have a variety of active functions such as anticancer, antioxidative, antimicrobial, and anti-inflammation (Cheng et al. 2011; Li et al. 2008; Fang et al. 2016; Benavente-García and Castillo 2008).

In the current study, we investigated the effects of several flavonoids on the metabolites produced by *Monascus aurantiacus* Li AS3.4384 (MAL) during liquid medium fermentation. Further, we analyzed the activity and structure–activity relationship between the flavonoids and metabolites secreted by MAL.

## Materials and methods

### Materials, fungal strains, and apparatus

MAL, a high producer of citrinin, was purchased from the Microbial Preservation Center (Beijing, China). Flavonoids, including genistein (purity 97%), daidzein (purity ≥ 98%), genistin (purity ≥ 95%), myricetin (purity 97%), and luteolin (purity ≥ 98%), were obtained from Aladdin Reagent Co. Ltd (Shanghai, China). Apigenin (purity ≥ 97%), baicalein (purity 98%), quercetin (purity 97%), and kaempferol (purity 97%) were purchased from Shanghai Macklin Biochemical Technology Co., Ltd (Shanghai, China). Pearl rice (produced in Heilongjiang) was obtained from a local supermarket. After grinding, the rice powder was filtered through a 100 mesh sieve. Methanol, acetonitrile (HPLC grade), and other reagents (analytical grade) were purchased from Aladdin Reagent Co., Ltd. (Shanghai, China). A spectrophotometer was purchased from Thermo Fisher Scientific (Oy Ratastie 2, FI-01620 Vantaa, Finland). KDC-140HR high-speed refrigerated centrifuge was obtained from Anhui Zhongke Zhongjia Scientific Instrument Co., Ltd. (Hefei, China). ZWY-2102C constant-temperature culture oscillators were purchased from Shanghai ZHICHENG Analytical Instrument Co., Ltd. (Shanghai, China). Electric thermostat blast drying oven was obtained from Huangshi Hengfeng Medical Instrument Co., Ltd. (Huangshi, China). Agilent 1260 high-performance liquid chromatography system equipped with an automatic injector, an UV–Vis detector, and a fluorescence detector was purchased from Agilent Technologies Inc. (Santa Clara, CA, USA). A digital thermostat water bath was obtained from Changzhou Langyue Co., Ltd. (Changzhou, China). YQ-620C ultrasonic cleaning instrument was purchased from Shanghai Yijing Ultrasonic Instruments Co., Ltd. (Shanghai, China). UPC-11-10T ultra pure water system was obtained from Wuhan Ulupure Pure Water Equipment Co., Ltd. (Wuhan, China).

### Liquid-state fermentation of MAL

MAL spores were prepared according to the method described in our previous study (Huang et al. 2019). The liquid-state fermentation of MAL was performed as follows: first, 1.0 mL of the spore solution (7.0 × 10^6^ spore/mL) was inoculated into a 250 mL Erlenmeyer flask with 100 mL seed culture medium (m/v) (3.0% glucose, 0.2% KH_2_PO_4_, 0.3% NaNO_3_, 0.05% KCl, 0.05% MgSO_4_·7H_2_O), and shaken at 180 rpm and 30 °C in a constant temperature incubator for 48 h. Subsequently, 5.0 mL of MAL seed solution was inoculated onto 100 mL aseptic liquid-state fermentation medium (m/v) (2.0% rice powder, 0.2% NaNO_3_, 0.05% KH_2_PO_4_, 0.1% K_2_HPO_4_, 0.1% MgSO_4_·7H_2_O), and rice powder inorganic salt medium with 0–5.0 g/L of genistein or other flavonoids at 30 °C, 180 rpm. The culture was incubated in a constant temperature shaking incubator. After completion of MAL fermentation, 1.0 mL of the fermentation broth was added to 2.0 mL methanol in a centrifuge tube, mixed well, and placed in a 60 °C water bath for 1 h, shaking constantly. After the fermentation broth was removed for cooling, the supernatant was taken and centrifuged at 8000 rpm for 15 min at 25 °C. After filtration through a 0.22 μm microporous organic filter for HPLC analysis.

### Effect of genistein and other flavonoids on biomass of MAL

Briefly, 5.0 mL of MAL seed solution was inoculated into a 250 mL Erlenmeyer flask with 100 mL of liquid-state fermentation medium supplemented with genistein or other flavonoids, along with a blank group, and fermented for 12 d at 30 °C in a constant temperature shaking incubator at 180 rpm. Each experimental and blank group was replicated at least three times. Next, the mycelium reached a constant weight and was measured.

### Effect of genistein and other flavonoids on pigment production by MAL

The liquid-state fermentation was carried out according to the above mentioned fermentation process. Genistein and other flavonoids were added to the culture medium and set with a blank control group. The three primary pigments were detected according to our previous study (Huang et al. 2019). Briefly, 1.0 mL of the fermentation broth was transferred into a 5.0 mL centrifuge tube. Following which 2.0 mL of methanol was added and mixed well by shaking. The centrifuge tube containing the sample was then placed in a water bath at 60 °C for 1 h. After removing the sample from the water bath and cooling, it was filtered with a 0.22 μm organic filter. Further, 100 μL was measured on a 96-well microtiter plate using a spectrophotometer. The yellow, orange, and red pigments were detected at 410 nm, 470 nm, and 510 nm, respectively. The measured result was multiplied by the dilution factor to obtain the desired pigment value. Additionally, the yellow pigment was determined by HPLC. The HPLC analytical conditions were as follows: a Thermo Syncronis C_18_ column (250 × 4.6 mm i.d., 5 μm) was used to determine the yellow pigment at 30 °C, using a UV–vis detector set at 410 nm. Mobile phase was acetonitrile/H_2_O (75:25, v/v), at a flow rate of 1.00 mL/min by isocratic elution, and automatic injection was set to 20 μL.

### HPLC and UPLC-QTOF-MS analysis

HPLC analysis was carried out at 30 °C, using a Thermo Syncronis C_18_ column (250 × 4.6 mm i.d., 5 μm) and a fluorescence detector (λex = 331 nm, λem = 500 nm). The mobile phase was acetonitrile/H_2_O (60:40, v/v), pH 2.6 (adjusted with phosphoric acid), at a flow rate of 1.0 mL/min by isocratic elution, and automatic injection was set to 20 μL. The UPLC-QTOF-MS analytical conditions were as follows. The UPLC was carried out on a Waters Acquity UPLC BEH C_18_ (100 × 2.1 mm i.d., 1.7 μm, Waters, Milford, USA) at 25 °C with a flow rate of 0.3 mL/min using an Agilent 1290 UPLC system (Agilent, Santa Clara, CA, USA). Mobile phase A contained water with 0.1% formic acid, and phase B consisted of acetonitrile. The gradient elution started with 10% B, linearly increased to 75% B in 20 min, and later on to 100% B in 0.1 min, kept constant for 2.9 min, and returned to 90% A in 0.1 min, followed by 2.9 min of re-equilibration. Agilent 6538 ESI-QTOF-MS was used, operating in the positive ion mode in the mass range of 60–1700 Da with the following mass spectrometer conditions: fragmentor = 130 V; collision energy = 60 eV; nebulizer gas = 40 psi; capillary voltage = 4500 V; drying gas flow = 10 L/min; gas temperature = 350 °C.

### Statistical analysis

The statistical analysis was carried out as described previously (Huang et al. 2019). All experiments were performed in triplicate (at least) and values were expressed as means ± standard deviations.

## Results

### Effect of adding genistein on citrinin production of MAL in different content rice powder medium

**Fig. 2:**
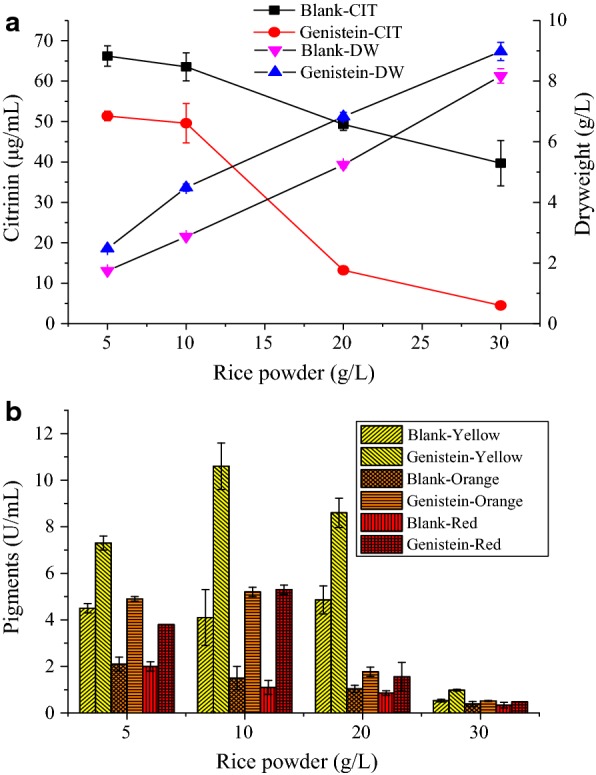
Effects of genistein (2.0 g/L) on **a** citrinin (CIT), biomass, and **b** pigments production by MAL in different content of rice powder in liquid-state fermentation medium at 30 °C, 180 rpm for 12 days. Significant differences of different the dots and the columns at p < 0.05

In this study, rice powder was used as a carbon source for the medium. During fermentation of *Monascus*, changes in carbon source resulted in important effects on secondary metabolites (Carels and Shepherd 1977; Wong et al. 1981). Optimizing the amount of the carbon source added is necessary for subsequent experiments. As shown in Fig. 2a, the citrinin content decreased as the added amount of rice powder increased. After adding genistein (2.0 g/L), citrinin showed a significant decrease trend compared with the blank group. When the amount of rice powder was 5.0 g/L and 10.0 g/L, the reduction rate of citrinin was about 22%. When the amount of rice powder increased to 20.0 g/L, the decrease of citrinin was more obvious, with a rate of about 80%; the reduction rates exceeded 88% when rice powder was added at 30.0 g/L. It was observed that the dry weight cells (biomass) increased as the amount of supplemented rice powder increased (Fig. 2a), suggesting that the addition of geinstein did not inhibit the growth of MAL (Fig. 2a).

It could be seen from the change of the pigments in Fig. 2b that the production of yellow pigment was highest for MAL, and the orange and red pigments were lower. As the amount of rice powder added increased, the yield of the three pigments gradually decreased. Among them, the content of the three pigments was similar when the amount of rice powder was 5.0 g/L and 10.0 g/L, and notable pigmentation was observed after adding genistein. When 20.0 g/L rice powder was added, the content of yellow pigment remained the same; however, the orange and red pigments decreased. The addition of genistein still promoted the production of *Monascus* pigment. When the amount of 30.0 g/L rice powder was added, the content of the three pigments was significantly reduced. Therefore, we found that when the amount of rice powder was increased, the pigment content was lowered. Combined with the addition of genistein, the citrinin had a better reduction effect and could improve the pigment yield.

### Growth curve of *Monascus* in rice powder medium

**Fig. 3:**
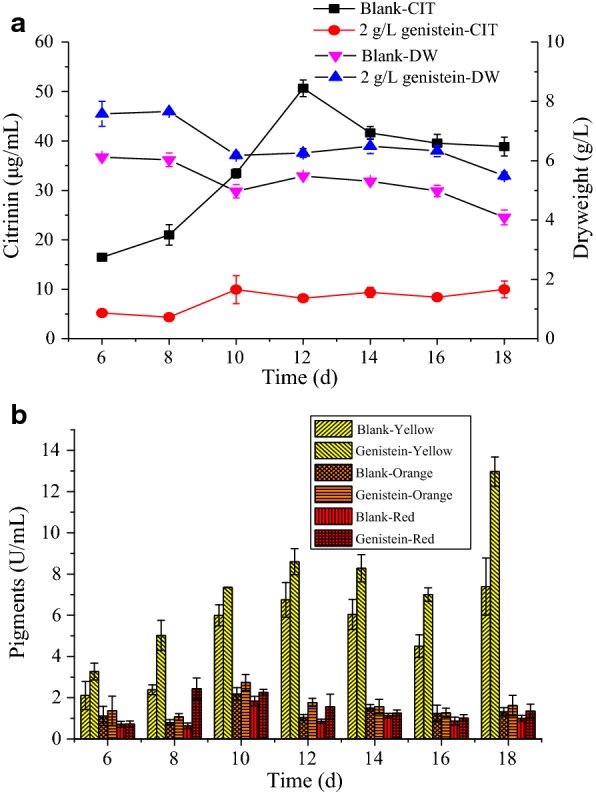
Effects of genistein (2.0 g/L) on **a** citrinin (CIT), biomass, and **b** pigments production by MAL at 30 °C, 180 rpm during different liquid-state fermentation time. Significant differences of different the dots and the columns at p < 0.05

As shown in Fig. 3a, a growth curve of citrinin produced by MAL fermented in rice powder inorganic salt medium. The yield of citrinin increased first, and it gradually became stable after reaching its highest point. The yield of citrinin was greatest at 12 days of fermentation. After the addition of 2.0 g/L genistein, the yield of citrinin was significantly reduced, which stabilized after reaching the highest value in 10 days. The reduction rate after genistein addition was the lowest at 12 days, approximately 80%. The growth trend of the dry weight of the bacteria was consistent with the blank group and genistein addition. Further, after genistein addition, it did not affect the growth stages of the cells, which could be seen from MAL staining in Fig. 3b. During the growth of MAL, the content of yellow pigment was significantly higher than orange and red pigments. When genistein was added, the three pigment contents were higher than those in the blank group. It could be seen that genistein had a significant effect on citrinin and promoted pigment growth.

### UPLC–QTOF–MS analysis of fermentation samples of different additions of flavonoids

The fermentation samples of different addition of flavonoids in the medium were analyzed by UPLC-QTOF-MS. The main compounds, including citrinin (ESI^+^ 251.0893), monascin (ESI^+^ 359.1836), ankaflavin (ESI^+^ 387.2151), and five unknown compounds (EIS^+^ 385.1995, 374.2720, 318.2980, 432.2351, and 302.3042), were found in the total ion chromatogram, and their retention times were 9.22 min, 16.24 min, 18.68 min, 3.57 min, 11.56 min, 11.75 min, 13.40 min, and 13.90 min, respectively (Fig. S1 and Fig. S2, Additional file 1). The results also showed that the different flavonoids had different effects on citrinin reduction in the fermentation samples, and genistein and baicalein markedly affected citrinin production. In addition, there were no new compounds generated in fermentation samples following flavonoid addition (Additional file 1: Fig. S1). The compounds retention time was 11.04 min (ESI^+^ 283.0648) and 10.36 min (ESI^+^ 359.1829) after adding baicalein and myricetin (Additional file 1: Fig. S1), respectively, and the two compounds were derived from raw materials.

### Structure–activity relationship analysis

**Fig. 4:**
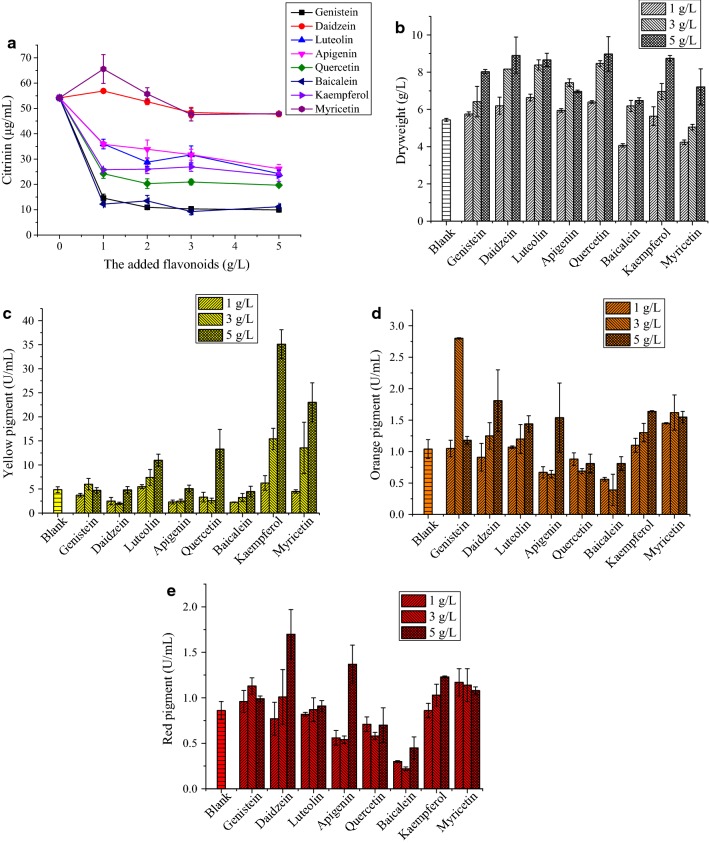
Effects of different types and quantities of flavonoids (1.0–5.0 g/L) on **a** citrinin, **b** biomass, **c** yellow pigment, **d** orange pigment, and **e** red pigment production by MAL in liquid-state fermentation at 30 °C, 180 rpm for 12 days. Significant differences of different the columns at p < 0.05

As shown in Fig. 4a, the effect of different amounts of flavonoids on citrinin reduction after liquid-state fermentation of MAL demonstrated different effects on citrinin. Overall, after reaching 2.0 g/L, the reduction in citrinin was relatively flat to constant. This might be related to the water solubility of flavonoids. Most of the flavonoids are poorly soluble in water; therefore, these are not fully utilized in the fermentation broth. Accordingly, 2.0 g/L was used in the experiment. Nine flavonoids had different abilities to reduce citrinin. Among them, daidzein and myricetin had the weakest effect. When the addition amount was 1.0 g/L and 2.0 g/L, daidzein or myricetin had no inhibitory effect on citrinin production by MAL. When the dosage was 3.0 g/L and 5.0 g/L, citrinin was reduced by about 10%. The reducing effects of genistein and baicalein on citrinin were similar. When 2.0 g/L genistein or baicalein was added, citrinin was reduced by about 80%; however, when baicalein was added, MAL barely produced pigments (Additional file 1: Fig. S3). When 2.0 g/L genistein was added, citrinin was reduced by about 60%. Because genistein is relatively expensive, the content optimization experiment was not performed. When 2.0 g/L of apigenin, luteolin, kaempferol, and quercetin were added to the medium, the content of citrinin was reduced by 46.9%, 45%, 51.1%, and 66.7%, respectively. When the dosage of these flavonoids was 3.0 g/L and 5.0 g/L, the effect on citrinin reduction was similar to 2.0 g/L flavonoid addition. The change of MAL biomass can be seen in Fig. 4b. Overall, the biomass increased with increase in flavonoid content, and only baicalein and myricetin had lower biomasses. The amount of biomass reflects the growth of MAL. Adding baicalein and myricetin might inhibit MAL growth, while the other six flavonoids did not affect growth. Figure 4c–e showed the variation of the contents of *Monascus* yellow, orange, and red pigments. Overall, with the increase in flavonoids, the pigment contents increased. For yellow, orange, and red pigments, the yield of baicalein was lower than the blank group. It is concluded that baicalein did not promote pigment production while reducing citrinin. Genistein had a higher ability to produce pigments than other flavonoids (Table 1). Therefore, the effect of genistein on the MAL fermentation was the best.

**Table 1:** The content of two yellow pigments (monascin and ankaflavin) in liquid fermentation samples after adding different flavonoids, as detected by HPLC (n = 6)

Experimental group	Monascin (μg/mL)	Ankaflavin (μg/mL)
Blank	20.69 ± 1.69	7.25 ± 0.74
Genistein	28.37 ± 2.72	8.50 ± 0.36
Daidzein	18.99 ± 2.62	5.53 ± 0.61
Luteolin	22.52 ± 2.28	6.71 ± 1.30
Apigenin	12.15 ± 2.76	6.71 ± 1.33
Quercetin	26.89 ± 1.17	7.79 ± 0.35
Baicalein	–	–
Kaempferol	18.97 ± 2.63	6.10 ± 1.26
Myricetin	32.06 ± 5.37	8.94 ± 0.08
Genistin	11.36 ± 5.17	3.52 ± 2.08

“–” means no detection

## Discussion

In this study, different rice powder contents exerted specific effects on citrinin production. When adding genistein and the same amount of rice powder, citrinin could be reduced evenly. Adding 2.0 g/L genistein to the culture medium had the best effect on citrinin reduction, and the content of pigments was also increased. However, the pigment content was the highest under the conditions of low rice powder content. This is possibly because the growth cycle of MAL was shortened due to low rice powder content, which makes the fungus produce more secondary metabolites. Citrinin and pigment yields were very high at low rice powder content of the medium. It has been previously found that the increase in rice powder content reduces dissolved oxygen (Hajjaj et al. 1999; Yang et al. 2015) in shaking flasks, slowing the growth of mycelium and reducing the production of secondary metabolites of MAL. After adding genistein, the growth curve of MAL was also affected. After 12 days of fermentation, citrinin was markedly reduced. After exploring the effects of genistein on metabolites of MAL, we further studied the effects of different kinds of flavonoids with different contents on metabolites of MAL. The structures of these flavonoids are shown in Fig. 1b, which were expressed as flavones, isoflavones, and flavonols.

From Fig. 4, it can be observed that the effects of flavonoids on the metabolites of MAL were different. Different flavonoids exerted different effects on citrinin and pigment. However, it can be seen that the greatest effect of the flavonoids on citrinin reduction at the addition amount of 2.0 g/L almost did not affect cell growth. Therefore, we used the addition of 2.0 g/L flavonoids for these studies.

Among the nine flavonoids, daidzein and myricetin showed the weakest effect on citrinin; however, genistein and baicalein had the best effect. These nine flavonoids are similar substances; therefore, comparing their structural differences could help us to further understand the relationship between flavonoids and the structure–activity effect on citrinin production by MAL. According to the structural formula, genistein and daidzein differ only at the C5-OH position (Fig. 1); however, the effect of genistein and daidzein on citrinin was quite different. Genistein significantly reduced citrinin content of MAL, but daidzein did not. We speculated that the C5-OH of ring A is the key site for citrinin reduction. Fischer et al. (2012) studied the effects of genistein and daidzein on the immunity of *Caenorhabditis elegans*. It was concluded that genistein decreased vitellogenin expression, but daidzein increased vitellogenin expression, which showed different trends, which may be related to C5-OH. This result was consistent with that of the current study. Poulsen et al. (2009) had reported the effects of genistein and daidzein on a series of physiological indicators in ovariectomized rats. Their results showed that Ileal and fecal digestibility of genistein was significantly greater than that of daidzein. The authors suggested that the difference in metabolism and structure may be responsible for their differences, and further verified that 4-ethylphenol was the main metabolite of genistein in rats. Djiogue et al. (2009) reported that genistein containing C5-OH had stronger binding ability to the estrogen receptor than daidzein, which further proved that C5-OH was an active site of flavonoids. Fang et al. (2019) found that C3-OH, C5-OH, C3′-OCH_3_, and C4′-OCH_3_ had good activity for the binding of flavonoids to P-Glycoprotein. Liu et al. (2012) studied the inhibitory effect of polymethoxyflavones (PFM) on *Aspergillus Niger* in citrus. The structure–activity analysis showed that flavones containing the C5-OH group had the strongest antifungal activity, which proved that C5-OH was the active group of flavonoids. Interestingly, in our study, genistein could reduce citrinin content in liquid-state fermentation of MAL, while daidzein had little effect on citrinin (Fig. 4a). Additionally, genistein had no effect on the biomass of MAL (i.e., it did not inhibit the growth of MAL) and could increase pigment production (Fig. 4b). It further indicated that genistein C5-OH played a key role on citrinin reduction during liquid-state fermentation of MAL.

Apigenin is an isomer of genistein, mainly in different substitution positions of the C ring. The results showed that apigenin reduced citrinin by about 50%, and genistein reduced citrinin by about 80%. From this, we could infer that the benzene ring at the C3 position can reduce citrinin production more effectively. This should be a secondary site of citrinin reduction, and the B-ring C4′-OH may also be an active site. Larrosa et al. (2006) mentioned that the interaction between C5-OH and C4′-OH at C3 facilitates genistein binding to phytoestrogen receptors with greater affinity than daidzein. Zhang et al. (2018) pointed out that hydrophobic groups and hydrogen bonds were important factors to stabilize the binding ability of flavonoids. For estrogenic flavonoids, methylation of hydroxyl group can reduce estrogenic activity; therefore, it is presumed that the hydroxyl group is necessary for flavonoids to possess estrogen. Similarly, the presence of these hydroxyl groups also had an important effect on citrinin reduction.

The structure difference of apigenin and luteolin lies in the hydroxyl number of the B ring. The results showed that apigenin and luteolin reduced citrinin by approximately 50–60%. It was concluded that the number of hydroxyl groups attached to benzene on the B ring did not affect the citrinin content. Kaempferol, quercetin, and myricetin also have structural similarities. They are all flavonols, containing C3-OH. On the B ring, the three hydroxyl groups are connected to 1, 2, and 3 hydroxyl groups, respectively. The citrinin reduction rate of quercetin was about 67%, and that of kaempferol was about 50%. Myricetin had no effect on citrinin. Because the myricetin solution was acidic and its pH was low during fermentation, it might promote citrinin (Lee et al. 2007). Therefore, we speculated that the ortho-hydroxyl group had a better effect on citrinin reduction for flavonols. For baicalein, there is a hydroxyl group at the C6 position, and the difference of its structure might influence the metabolites of MAL. The results showed that baicalein significantly reduced citrinin production, which was similar to genistein, but the pigments were greatly reduced. During the fermentation process, we also found that the morphology of MAL changed in the medium supplemented with baicalein. Therefore, we speculated that the addition of baicalein might completely change the metabolic pathway of MAL. This requires further exploration in future studies. In addition, studies have shown that C7-OH is also an active site of flavonoids (Echeverry et al. 2010). Comparison with adding genistein and genistin to the medium, the results showed that genistein with C7-OH had better effect on citrinin reduction.

In the study of nine flavonoids, genistein strongly inhibited citrinin production and improved pigment contents. Therefore, C5-OH and C4′-OH markedly inhibited citrinin production by MAL. C5-OH played a dominant role in reducing citrinin. Benzene C4′-OH on the C3 ring also played a role in inhibiting citrinin production; thus, there might be some interaction between them. Wu et al. (2013) also reported that flavonoids exhibited good antimicrobial activity against E. coli, including C5-OH and C4′-OH in ring A and B. Xiao et al. (2011a, b) proposed that the hydroxylation of flavonoids had an important effect on the covalent binding of human blood and inhibition of xanthine oxidase activity in bovine blood. Simons et al. (2005) suggested that the degradation of flavonoids by human microorganisms was also related to their structure. The C4′-OH was very important for rapid microbial degradation, but only existed in the presence of C5-OH. The presence of methoxy groups in A or B rings makes flavonoids degradable against microorganisms. Flavonoids with C5-, C7-, and C4′-hydroxyl groups degraded faster than flavonoids lacking all these hydroxyl groups in all three species. Therefore, hydroxylation of specific sites of flavonoids was necessary for their biological activity expression, and it could well explain that flavonoids could reduce citrinin production by liquid-state fermentation of MAL.

To the best of our knowledge, this is the first study investigating the, structure–activity relationship of flavonoids on citrinin reduction by *Monascus* during liquid-state fermentation. Genistein and other flavonoids could affect the citrinin and pigments production by MAL. The C5-OH, C4′-OH, and C7-OH of flavonoids might play a key role for their effects on citrinin reduction in liquid-state fermentation of MAL. The mechanism of flavonoids on metabolites of *Monascus* calls for further study of its intrinsic genomics, proteomics, and metabonomics.

## Supplementary information

**Additional file 1.Fig.S1**. Total ion chromatogram of UPLC-QTOF-MS of the liquid fermentation samples following addition of different flavonoids and blank group. 2-citrinin, 7- monascin, 8- ankaflavin. **Fig. S2** QTOF-MS of several main compounds in the liquid fermentation samples following addition of different flavonoids. **Fig. S3** The liquid fermentation samples following addition of different flavonoids and blank group.

## Data Availability

We conducted experiments and data generated. All data is shown in figures, tables and additional data.
