# Supplementary material for: Effects of some flavonoids on the mycotoxin citrinin reduction by Monascus aurantiacus Li AS3.4384 during liquid-state fermentation
Source: AMB Express. 2020 Feb 3;10:26. doi: 10.1186/s13568-020-0962-7 (PMC6997324; doi:10.1186/s13568-020-0962-7)
Supplement: Supplementary file 1 — Additional file 1.Fig.S1. Total ion chromatogram of UPLC-QTOF-MS of the liquid fermentation samples following addition of different flavonoids and blank group. 2-citrinin, 7- monascin, 8- ankaflavin. Fig. S2 QTOF-MS of several main compounds in the liquid fermentation samples following addition of different flavonoids. Fig. S3 The liquid fermentation samples following addition of different flavonoids and blank group. [file 13568_2020_962_MOESM1_ESM.docx]

**Additional materials**

**AMB Express**

**Effects of some flavonoids on** **the mycotoxin citrinin reduction by *Monascus* during liquid fermentation**

Yanling Wang ^a,b^, Heng Gao ^a,b^, Jianhua Xie ^a^, Xiujiang Li ^c^, Zhibing Huang ^a,b,^*

^a^ State Key Laboratory of Food Science and Technology, Nanchang University, No. 235 Nanjing East Road, Nanchang 330047, China

^b^ Sino–German Joint Research Institute, Nanchang University, No. 235 Nanjing East Road, Nanchang 330047, China

^c^ The First Affiliated Hospital of Nanchang University, Nanchang University, No.17 Yongwai Main Street, Nanjing West Road, Nanchang 330006, China

Running head: Genistein and other flavonoids affects citrinin production by *Monascus*

*To whom correspondence should be addressed. (Telephone: +86-0791-88305177-8109; Fax: +86-0791-88333708; E-mail: hzbchem@ncu.edu.cn).

**Figure Captions**

**Fig.S1.** Total ion chromatogram of UPLC-QTOF-MS of the liquid fermentation samples following addition of different flavonoids and blank group. 2-citrinin, 7- monascin, 8- ankaflavin.

**Fig. S2** QTOF-MS of several main compounds in the liquid fermentation samples following addition of different flavonoids.

**Fig. S3** The liquid fermentation samples following addition of different flavonoids and blank group.

**Fig. S1**

**
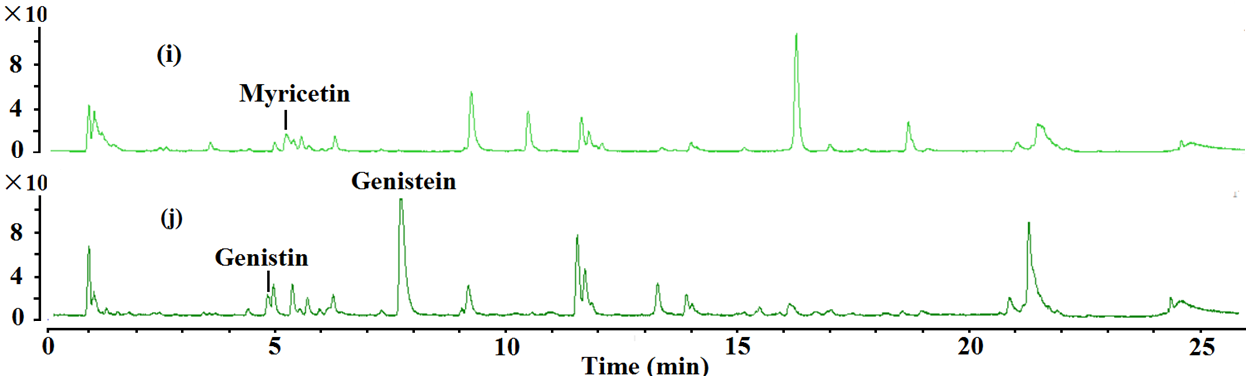

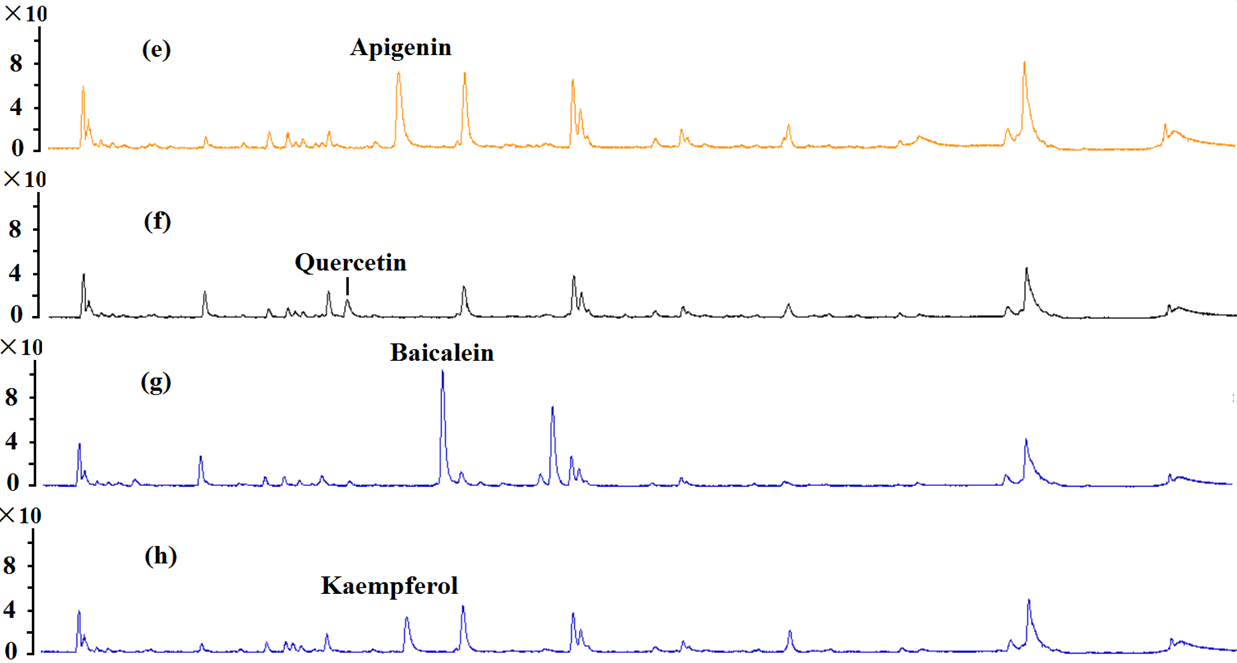

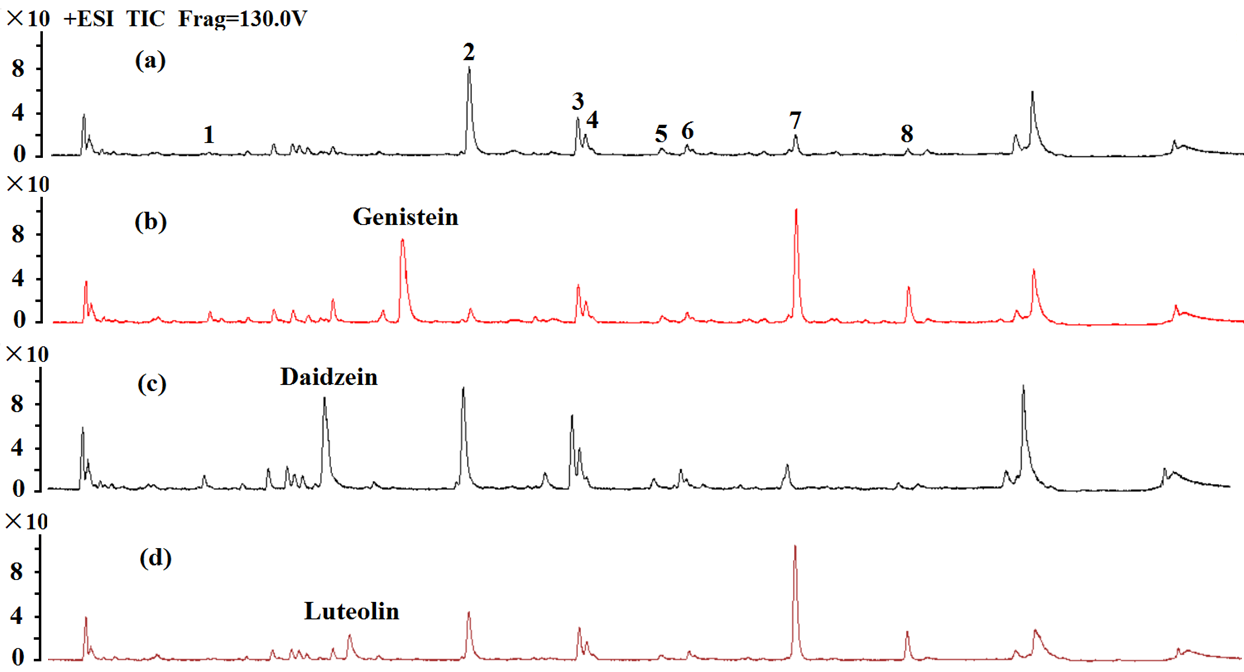
**

**
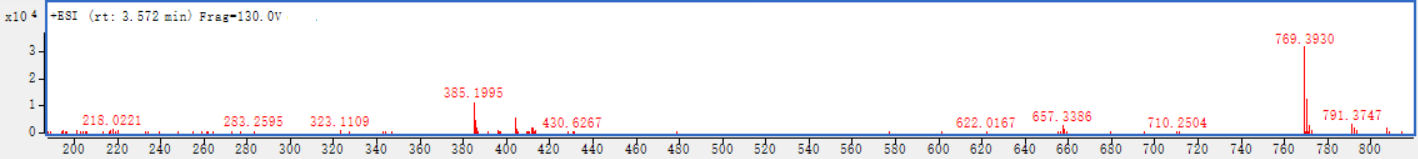
Fig. S2**


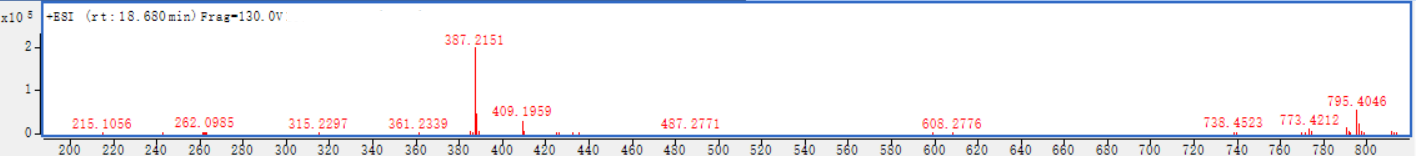

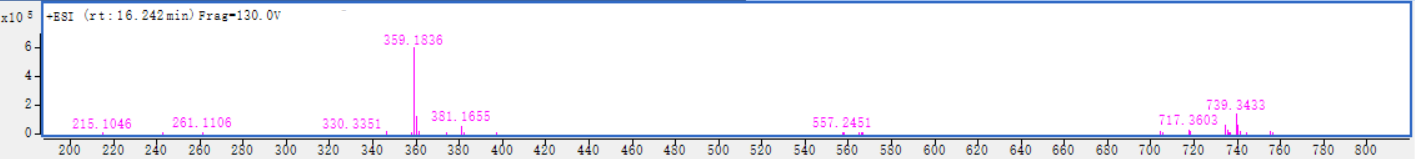

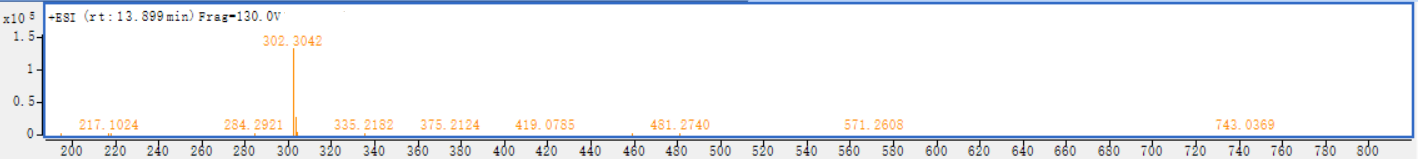

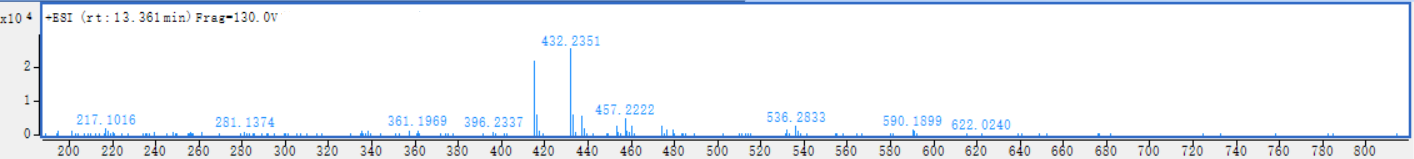

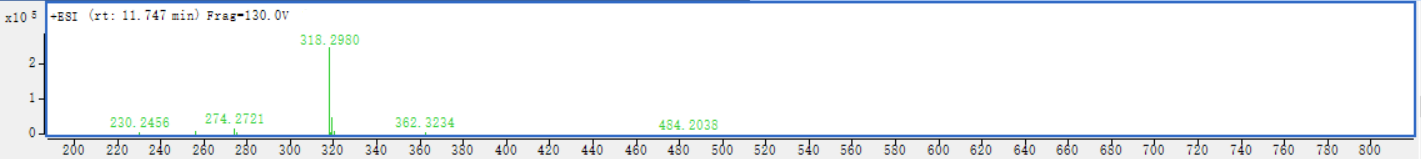

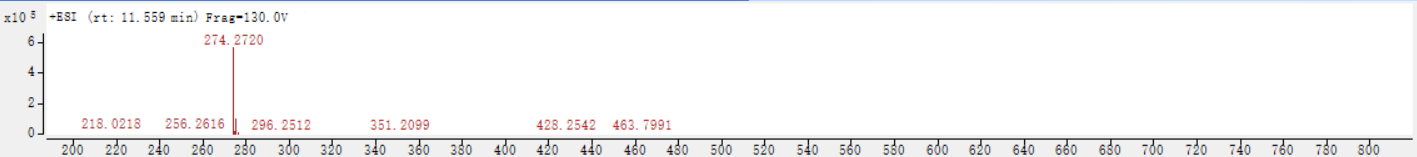

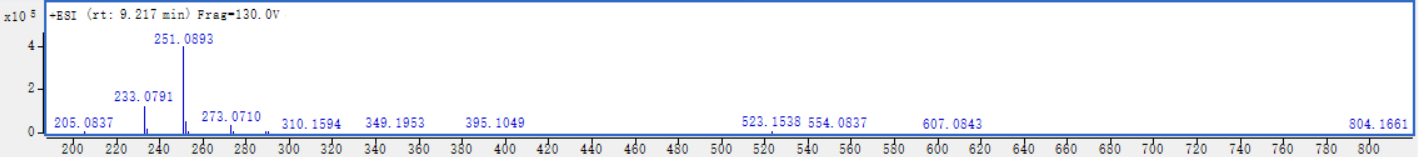

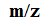


**Fig. S3
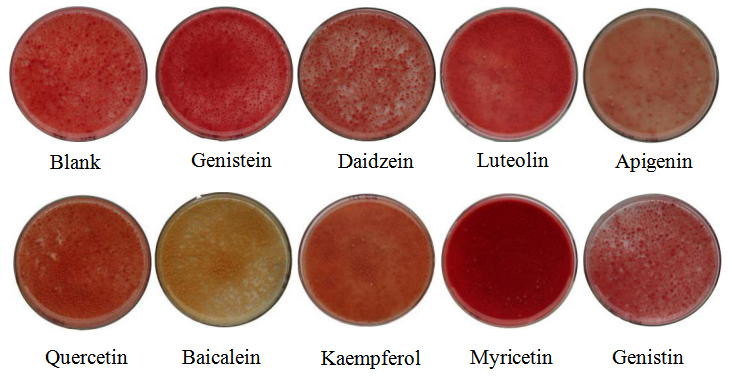
**
